# Rehabilitation Interventions to Promote Recovery from Schizophrenia: A Systematic Review

**DOI:** 10.3389/fpsyt.2017.00100

**Published:** 2017-06-12

**Authors:** Laurent Morin, Nicolas Franck

**Affiliations:** ^1^Resource Center of Psychosocial Rehabilitation and Cognitive Remediation, Le Vinatier Hospital, Lyon, France; ^2^Centre Hospitalier Le Vinatier, Lyon, France; ^3^UMR 5229 CNRS & Claude Bernard University, University of Lyon, Lyon, France

**Keywords:** schizophrenia, recovery, cognitive remediation, cognitive-behavioral therapy, psychoeducation, functional outcomes

## Abstract

Only one out of seven patients recovers after a first episode of psychosis despite psychiatric care. Rehabilitation interventions have been developed to improve functional outcomes and to promote recovery. We conducted a systematic review of the effectiveness of the main psychiatric rehabilitation interventions following a search of the electronic databases Pubmed, ScienceDirect, and Google Scholar using combinations of terms relating to cognitive remediation, psychoeducation, cognitive-behavioral therapies, and schizophrenia. Eighty articles relevant to the topic of interest were found. According to results, cognitive remediation has been found to be effective in reducing the impact of cognitive impairment, social skills in the learning a variety of skills and to a lesser extent in reducing negative symptoms, psychoeducation in improving compliance and reducing relapses, and cognitive therapy in reducing the intensity of or distress related to positive symptoms. All psychosocial rehabilitation interventions should be considered as evidence-based practices for schizophrenia and need to become a major part of the standard treatment of the disease.

## Introduction

Recovery from mental illness can be defined in two different ways. On the one hand, psychiatric consumers define recovery as the attainment of a meaningful and valued life, rather than the absence of symptoms (1) while on the other, psychiatrists have developed a “medical” model of recovery placing the emphasis on elimination of symptoms and return to normal functioning (2). The latter view is nearer to the concept of remission and is based more on objective criteria.

In the literature, the lack of consensus on the definition of recovery gives rise to heterogeneous data with the proportion of people with schizophrenia achieving recovery varying from 13.5 to 50% (3). Since recovery is a multidimensional concept, some authors suggested that relevant indicators should consider at least two areas: clinical remission and social functioning. The results of one recent meta-analysis using these criteria (3) were less optimistic than those of previous works: the proportion of individuals with schizophrenia who met the criteria for recovery and appeared stable over time was only 13.5%. This suggests that functional outcomes are undoubtedly impaired in schizophrenia and should be a priority target for therapeutic interventions (3).

A large body of literature has studied the factors that may affect these functional outcomes. Neurocognition is one of the first factors described. Early studies showed that neurocognitive variables were significantly related to functional outcomes, accounting for approximately 25–50% of the variance in real-world functional outcomes (4, 5). Other variables such as intrinsic motivation and metacognition are also mentioned in few studies and may serve as mediators between neurocognition and functional outcomes (6, 7). To better explain causal pathways, researchers have built sophisticated models with parameters such as functional capacity, social cognition, and symptoms to take into account the complexity of the functioning.

Functional capacity is defined as the ability to perform tasks relevant to everyday life in a structured environment guided by an examiner. This includes the aptitude to perform in the field of residential functioning, work, and social skills (8). Several works have shown that functional capacity is at least as strongly correlated with real-world functional outcomes as cognitive performance (8, 9). Recent studies have revealed that the impact of cognitive impairment could be mediated by functional capacity (4, 10).

Social cognition is a multidimensional construct that comprises emotional processing, social perception and knowledge, theory of mind and attributional biases. According to most studies, social cognition probably also mediates the effect of neurocognitive impairment on real-life functioning (10, 11). A meta-analysis showed that social cognition may have a stronger impact on variance in community outcome (16%) than neurocognition (6%) (11).

Symptoms have been associated with functional outcomes from the beginning with negative symptoms appearing to interfere more than positive ones (12). Both direct and indirect relationships between negative symptoms and real-life functioning have been reported (13). They seem to mediate the impact of variables such as neurocognition or functional capacity on real-world functioning (9). It appears that symptoms such as amotivation and avolition have the greatest impact (13).

Most recent works confirm these findings and also refer to additional variables more connected with the patients’ environments. A study that involved a large sample of patients with schizophrenia (*n* = 921) summarized variables affecting real-life functioning and pooled them into three categories: variables related to the disease (cognition, symptoms, and functional capacities), variables linked to personal resources (resilience and engagement to services), and variables related to the context in which the person lives (internalized stigma and social support). The study showed that resilience, stigma, and engagement with mental health services mediate the relationships between symptomatology, cognition, and real-world functioning (13). Another recent work showed that negative symptoms predict social deficits but not impairment in everyday activities and vocational outcomes contrary to cognition and functional capacity (14).

**Figure 1:**
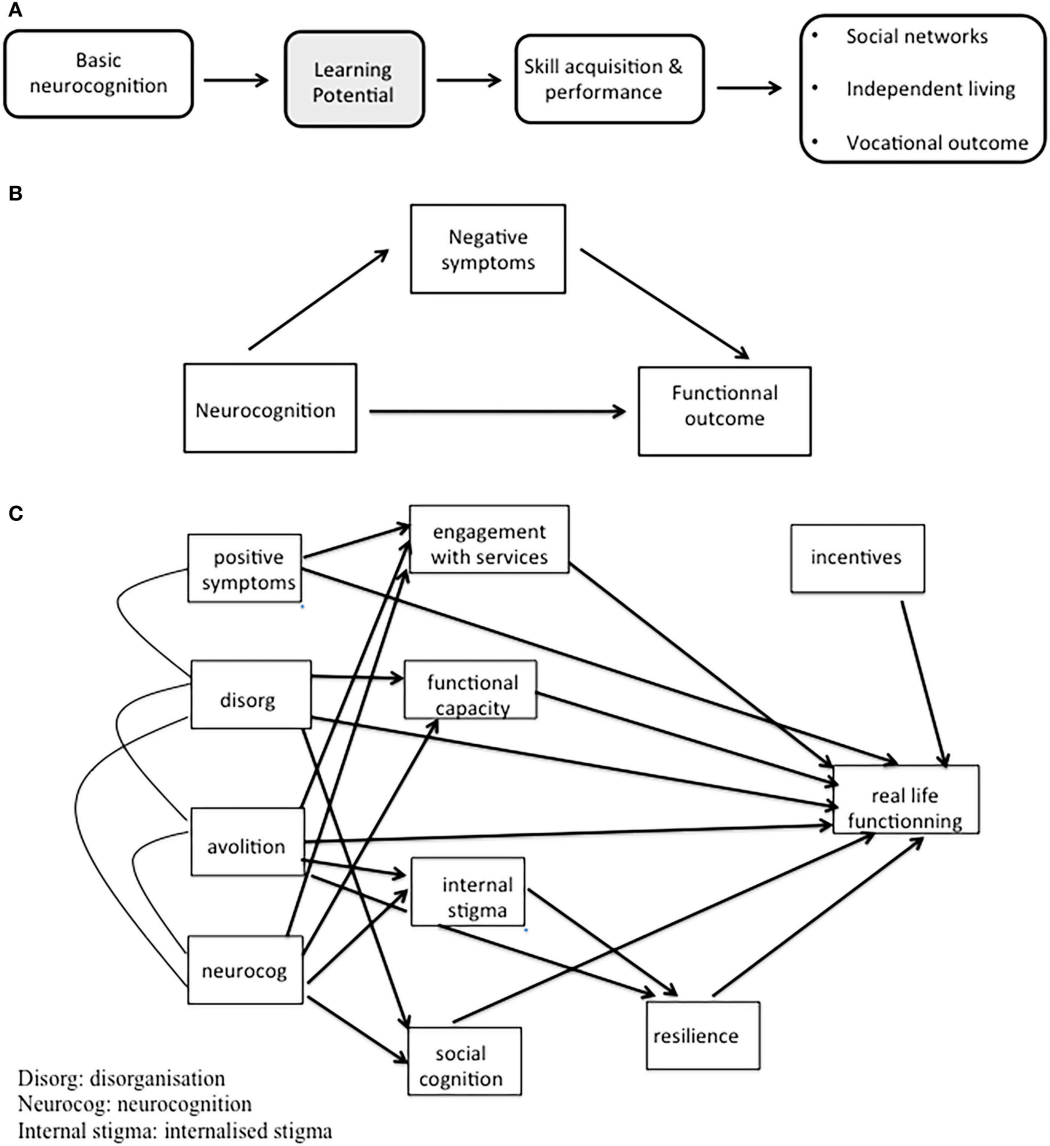
Evolution of models explaining real-world functioning in schizophrenia. **(A)** Adapted from Green et al. (4). **(B)** Adapted from Ventura et al. (12). **(C)** Adapted from Galderisi et al. (13).

Models explaining real-world functioning have become increasingly complex over time, with an exponentially growing number of factors. Some authors propose a single pathway, while others, like Galderisi, suggest multiples pathways (13). Hence, the question of one versus multiple pathways to outcomes in schizophrenia is not yet settled (15). Figure 1 summarizes this evolution. Most of the models cannot explain more than 50% of the functional outcome variance, which means that more variables should be taken into account in the prognosis of severe mental illnesses.

Two suggestions can be made based on these data. First, various factors need to be assessed to establish an individual “functional diagnosis.” Some factors are inherent to the patients (cognition, engagement with services, functional capacity, symptoms, resilience, and recovery processes), whereas others are related to their social context (internalized stigma, social support, resources, etc.). Such an assessment would help to determine an individualized intervention plan and to define life goals in collaboration with the patient.

Second, appropriate treatment targeting neurocognition, social cognition, negative symptoms and functional capacity, and integrative interventions combining different therapies need to be instituted taking into account the specific needs of each patient.

Rehabilitation or psychosocial interventions have been developed to complement psychotherapy and psychopharmacological treatments (16, 17). Indeed, drug treatments and supportive therapies do not have a specific effect on cognitive impairment, insight, social skills, and interaction disorders, whereas rehabilitation tools especially target these dimensions (17, 18). Rehabilitation interventions also share common values with the “subjective” model of recovery. Indeed, they promote taking an active position against the disorder, which encourages self-determination and empowerment.

Many tools can be used in the field of rehabilitation: case management, supported employment (SE), cognitive remediation, psychoeducation, and cognitive-behavioral therapies. In this review, we focused on rehabilitation interventions that particularly target the dimensions quoted above. Thus, we studied the three following interventions: (1) cognitive remediation, (2) psychoeducation, and (3) cognitive-behavioral therapies. Each type of intervention has different targets, but each favors functional recovery.

### Cognitive Remediation

Cognitive remediation for schizophrenia is “a behavioral training-based intervention that aims to improve cognitive processes (attention, memory, executive function, social cognition or metacognition) with the goals of durability and generalization” (Cognitive Remediation Experts Working Group, 2012) [c.f. Ref. (19)]. Cognitive remediation therefore aims to limit the impact of cognitive impairment on everyday functioning (20).

Cognitive disorders are very common: four out of five patients suffering from schizophrenia display cognitive impairment (21). Moreover, cognitive disorders are a major determinant of functional disability. Since cognitive impairment is very variable in schizophrenia, a neurocognitive assessment should be proposed to all patients to define their cognitive profiles, determine the functional repercussions of the cognitive disorder, and identify their cognitive strengths and weaknesses (21). An assessment of social cognition is also essential (11).

### Psychoeducation

The psychotic experience often leads to feelings of inconsistency and loss of direction. In the early course of the disease, people often feel like they are passive victims of schizophrenia as they lose their sense of personal efficacy and their hope in recovery.

Psychoeducation is defined as a “systematic, structured, didactic information on the illness and its treatment, and includes integrating emotional aspects in order to enable patients or family to cope with the illness” (22). It features common structural components since each program is designed and led by health professionals. A collaborative relationship is established between the mental health professionals and the patients or their families, to help the latter to share the burden of the illness and work toward the patients’ recovery (23). The core elements of psychoeducation programs are information about the signs and symptoms of schizophrenia, relapse prevention, and treatment of psychosis. Another important goal is to help patients to find a meaning to their illness and to adopt a constructive attitude toward their experience of psychosis. Psychoeducation cannot be described as the simple transmission of information; it places people with schizophrenia in a position where they take action (24). Psychoeducation should provide patients with information about the illness and its treatment as well as disease management problem-solving and coping skills and on how to access community mental health-care services, the purpose being to help patients better cope with the disease (22).

Family intervention shares a number of similarities with patient psychoeducation. It provides relatives with information about the nature, symptoms, and diagnosis of the disease to help them to identify its possible manifestations. It underlines that psychosis may be exacerbated by stress or substance use, helps identify signal symptoms announcing a relapse and explores the effect of pharmacological and psychosocial treatments. Family intervention focuses on improving both patient and family outcomes, i.e., on reducing the burden of disease (24).

Psychoeducation aims to help patients and their families understand the disease and treatment, cooperate with caregivers, live healthier lives, and maintain or improve their quality of life; consequently, it has an impact on several functional determinants, such as service engagement (active participation in defining treatment plans, ability to seek service help if needed, etc.), resilience, and self-stigma.

### Cognitive-Behavioral Therapy

Cognitive-behavioral therapies (CBT) are an essential part of non-pharmacological interventions for schizophrenia. They constitute a heterogeneous group of therapies sharing common features (Box 1) (25) with the main techniques used being social skills training and cognitive therapy (CT).

Box 1 **Principles of cognitive-behavioral therapies.**

– Modification of behavior and/or content of dysfunctional thoughts based on learning theory and data from experimental psychology;
– Collaborative approach: the patient plays an active role in the therapy;
– Priority is given to the experiences, needs, and demands of the patient;
– Therapeutic alliance;
– Goals for therapy defined in consultation prior to beginning treatment;
– Short and defined duration.

#### Social Skills Training

Social skills consist in three main components: receiving skills (social perception), processing skills (social cognition), and sending skills (behavioral responding or expression) (26, 27). Lack of social skills is one of the major deficits among people with schizophrenia. Impaired social skills significantly reduce patient autonomy and may lead to social withdrawal or isolation (28).

Behavioral treatment of schizophrenia is primarily based on the acquisition of new social interaction modalities. Social skills training is rooted in operant conditioning and learning theory (28). It is based on behavioral therapy principles and techniques for teaching individuals to communicate their emotions and requests so that they are more likely to achieve their goals and meet their needs (28). Although social skills training programs differ in implementation setting, duration and content, they all use a similar approach for teaching skills, including goal setting, role modeling, behavioral rehearsal, positive reinforcement, corrective feedback, problem-solving techniques, and home assignments to practice skills and promote generalization (29). Patients are usually given social skills training in groups led by two therapists. Training patients in a group provides an opportunity for self-help and peer support and enables participants to learn from each other’s real-life experiences and efforts at problem solving (28).

Social skills training targets social, independent living skills and thus probably has an impact on factors such as social cognition, functional capacity, or symptoms.

#### Cognitive Therapy

Reasoning and attributional biases, including jumping to conclusions and lower belief flexibility, are well described in psychosis (30). People experiencing psychosis are more likely to exhibit a personal, external attributional style. CT for psychosis aims at modifying dysfunctional beliefs by helping people to understand the links between perceptions, beliefs, and emotional and behavioral reactions (31). It allows the patient to question evidence supporting his/her beliefs and brings them to self-observe, to record their thoughts and behaviors, and to explore various coping strategies (31). Patients learn to cope with psychotic symptoms not controlled by medication and to reduce their impact on everyday life using structured techniques (Box 2).

Box 2 **Techniques used in cognitive therapy (31).**

– Education about the disease;
– Normalization of psychotic symptoms;
– Application of symptom-management techniques;
– Questioning of evidence underlying beliefs;
– Engagement in reality testing.

Initially, work in CT for psychosis targeted positive symptoms but recently, greater attention has been focused on negative symptoms. Cognitive models of negative symptoms have been conceptualized as maladaptive strategies aiming to protect individuals from expected pain associated with engagement in constructive activity. Treatment of negative symptoms uses the same techniques as those used for positive symptoms; in this case negative symptoms are conceptualized as negative self-beliefs (31). CT may be an efficient way to reduce the functional impairment associated with symptoms.

We conducted a systematic review of the literature for four of these treatments targeting effectiveness: cognitive remediation, psychoeducation, social skills training, and CT. Specific attention was paid to the functional effects of the treatments.

## Method

### Search Strategy

Electronic databases (PubMed, ScienceDirect, and Google Scholar) were searched for studies published in English between 1995 and 2017 that examined the effects of cognitive remediation, psychoeducation, and cognitive-behavioral therapies. After experimentation, the following terms were defined and searched for in the screening: (“schizophrenia”) AND (“cognitive remediation” OR “psychoeducation” OR “family psychoeducation” OR “social skills training” OR “cognitive behavior therapy”). To ensure no important review was overlooked, we proceed with an additional search using the terms (“schizophrenia”) AND (“cognitive remediation”); (“schizophrenia”) AND (“psychoeducation”); (“schizophrenia”) AND (“Family psychoeducation”); (“schizophrenia”) AND (“social skills training”); (“schizophrenia”) AND (“cognitive behavior therapy”).

Since the literature on this subject is very abundant, we only selected review articles and meta-analyses. We especially focused on the effectiveness of each technique and on real-life functioning. The search was also limited to peer-reviewed journal articles.

A total of 331 articles were initially identified as potential candidates for inclusion. After an initial review by the first author, 125 articles were excluded from the analysis (72 duplicates, 53 in other languages).

We then independently assessed the remaining studies for inclusion or exclusion from the systematic review. One inclusion criterion was that the patients had to be adults (18+) with schizophrenia or a schizophrenia spectrum disorder. Studies based on samples including children or teenagers were excluded from the review. The full text of the manuscript had to be available. A total of 95 papers met the inclusion criteria and were eligible for the review; 111 articles were excluded from the analysis (30 referring to other diagnoses, 10 without the full text available, 71 on other topics).

The articles found to be relevant to the topic of interest (*n* = 95) were reviewed and checked for methodological rigor and validity by the two authors (Laurent Morin and Nicolas Franck); 34 papers were excluded from the analysis: in 12 articles, the topic was too specific—for example, “cognitive remediation in India,” in 14 other articles, the diagnoses were too heterogeneous, and in the last eight articles, the main topic was not the effectiveness of the rehabilitation tools.

**Figure 2:**
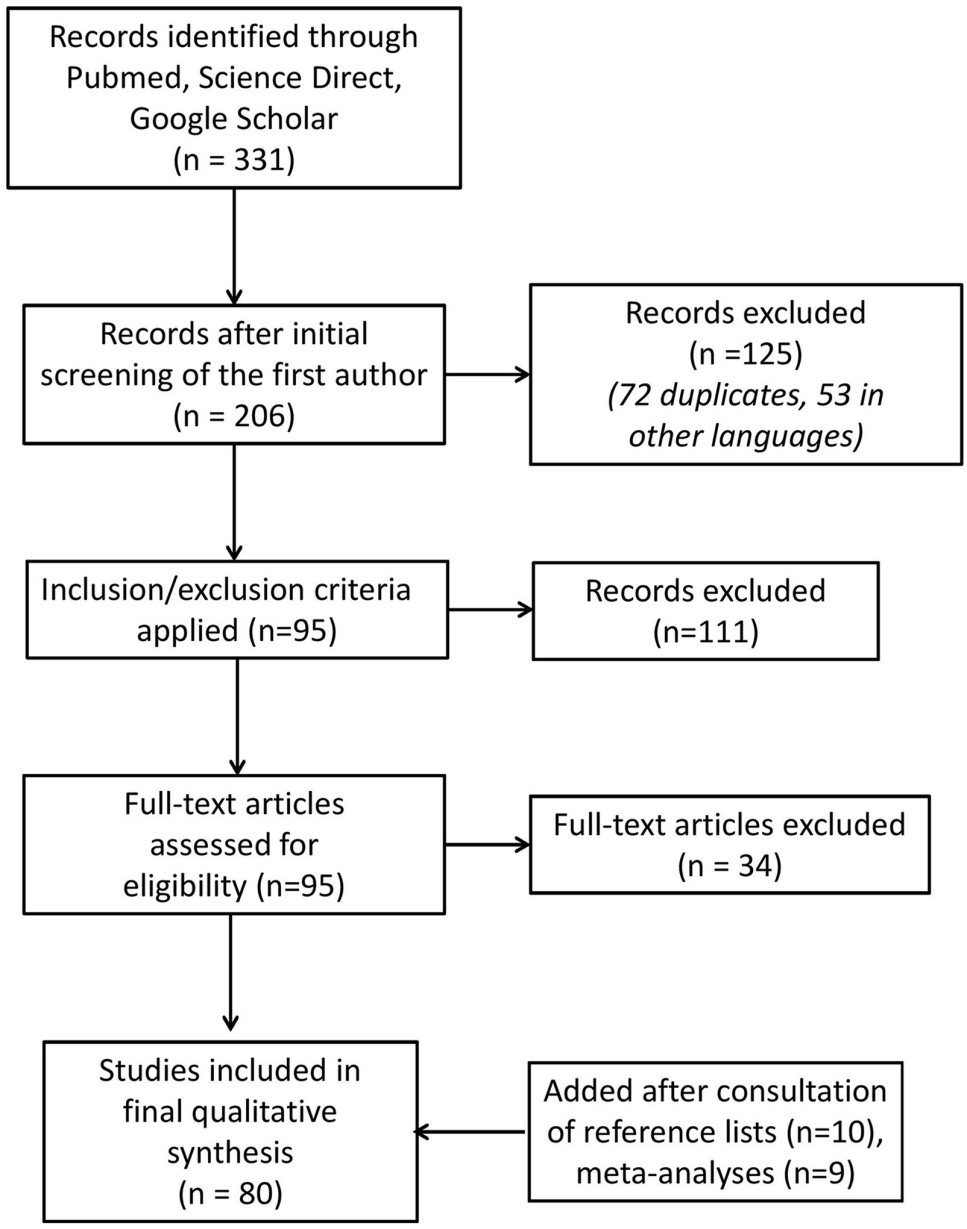
Study flow diagram.

All reference lists of the selected articles were also searched to identify further relevant trials: we added 10 more articles to the records. In order not to lose any other meta-analyses, we conducted a new search for each technique with the terms (“schizophrenia”) AND (“cognitive remediation”) AND (“meta-analysis”); (“schizophrenia”) AND (“psychoeducation or family psychoeducation”) AND (“meta-analysis”); (“schizophrenia”) AND (“socials skills training ”) AND (“meta-analysis”); (“schizophrenia”) AND (“cognitive behavior therapy”) AND (“meta-analysis”); nine more works were thus identified. Altogether, a total of 80 articles were finally reviewed in this work (Figure 2).

## Results

### Efficacy of Cognitive Remediation

Two meta-analyses (19, 32) showed the effectiveness of cognitive remediation in the management of neurocognitive disorders. Regarding effect size (ES), McGurk et al. showed that cognitive remediation had a significant impact on cognition with a medium ES (0.41) (32). The other meta-analysis (19) confirmed these results, demonstrating an overall ES of 0.45 for cognitive performance.

Cognitive remediation is also effective on psychosocial functioning. Two meta-analyses (19, 32) reported positive results with a small to medium ES (around 0.36) for social functioning and a small ES for symptoms (0.28). The impact of cognitive remediation on the patients’ ability to work is also positive (33). People who benefit from cognitive remediation work longer hours and have more opportunities to maintain work than people who do not (33, 34). Cognitive remediation is almost ineffective on symptoms. Even if patients with marked symptoms may improve their cognitive performances, the benefits of cognitive remediation are more significant in less symptomatic patients (19). A recent meta-analysis focused on the effect of cognitive remediation on negative symptoms. In this work, cognitive remediation was found to have a significant effect on negative symptoms (0.36) (35). Compared to the Wykes et al.’s meta-analysis (19), the effect was close to their ES of 0.18 for symptoms. As in the Wykes et al.’s meta-analysis, cognitive remediation programs associated with adjunctive psychiatric rehabilitation including psychoeducation and training to develop social, vocational, and daily living skills had a significantly more positive effect on functioning than cognitive remediation programs delivered alone (19, 35).

The effect of cognitive remediation seems generally homogeneous regardless of the method used (computer or paper-and-pencil tasks) and program duration (19, 36). Nevertheless, one study outlined the importance of adjusting the level of computerized exercises to the patients’ cognitive performances (37). Even if each cognitive remediation program is specific in terms of number of sessions, it seems that mild improvement may be observed with remediation of limited duration (from 5 to 15 h). Concerning the role of the patients’ age, results are more heterogeneous. Some authors suggest that younger people are more likely to benefit from cognitive remediation (38–40). More recent studies show that cognitive remediation in early psychosis has an impact on various aspects of schizophrenia such as cognition, functioning, and symptoms (41, 42). According to other works, cognitive remediation appears less effective in young populations than in patients with chronic conditions (43).

It seems that the majority of meta-analyses published in the last 5 years were adequate in terms of methodological quality; this is encouraging considering the concerns about the reliability of the results of cognitive remediation (44).

### Efficacy of Psychoeducation for Patients

The main criteria used to assess the efficacy of psychoeducation are relapse rate, decrease of symptoms, treatment adherence, knowledge of the disease, and functioning in the community. Most large-scale works on psychoeducation do not differentiate between information provided to the family from that provided to the patient. A Cochrane meta-analysis comparing the efficacy of psychoeducational interventions in schizophrenia to standard treatment in 10 randomized controlled trials (RCTs) showed that psychoeducational interventions significantly decreased relapse or readmission rates at 9–18 months follow-up compared with standard treatment (45). The authors estimated that 12 relapses could be avoided, or at least postponed, if 100 patients with schizophrenia received psychoeducation (45). Secondary outcomes such as knowledge gain and overall level of functioning indicated that psychoeducation had a positive effect on these dimensions (45).

Another meta-analysis including 18 studies showed the benefit of psychoeducation on relapse after 12 months (medium ES, 0.48) and on knowledge of the disease (medium ES, 0.48), but no effect on symptoms and on psychosocial functioning (46). A more recent review published in the Cochrane database and involving more than 5,000 patients (mostly inpatients) included in RCTs (*n* = 44) highlighted that psychoeducation improves patient compliance compared to standard treatments and reduces rate of relapse and hospitalization in the short term (6 months) (47). In these works, the median length of psychoeducation therapies was around 12 weeks, which is very costly in terms of time. Some works seem to indicate that short psychoeducation programs (<8 sessions) also reduce relapse and promote medication compliance, but these results have to be confirmed by further high-quality studies (48). It is also difficult to get a consistent view of the various measures of functioning as the data were very heterogeneous. For the global functioning outcome “no clinically significant improvement” was found, but in the medium term, the authors found that treating four people with psychoeducation instead of standard care resulted in one additional person showing improvement. Short-term and long-term data also favored the psychoeducation group, but results were not statistically significant. Overall, it seems that global functioning is helped by the psychoeducation approach (47).

Merinder’s review including seven studies confirmed an improvement of knowledge about the disease with small effects on adherence and relapse rates (49). A study quoted by several reviews showed that psychoeducation is effective in reducing readmission rates after 5 years in patients with medium duration illness (4–7 years) (24, 50). It seems that the content of psychoeducation programs needs to be adapted to the different stages of the disease. According to some authors, psychoeducation is especially adapted to patients in the early stages of disease when the content of the session tries to establish links with their own experience (50).

Psychoeducation could also play a major part in interventions to reduce internalized stigma. In a recent meta-analysis, psychoeducation was the most commonly used technique in controlled randomized studies on stigma intervention. This work could not statistically determine which interventions significantly reduced internalized stigma outcomes due to the scarcity of the studies; but most of the studies similarly found that psychoeducation and cognitive challenging were key components (51).

### Efficacy of Family Psychoeducation

A review on psychosocial treatment for schizophrenia showed that long-term family psychoeducation reduces the patients’ “vulnerability” to relapses over a period of 1–2 years (52). Other works mentioned the long-term effectiveness of psychoeducation (a combination of family and individual approaches). Patients suffering from schizophrenia benefiting of a brief eight-session psychoeducational program had significantly lower hospitalization rates after 12 and 24 months compared with standard treatment without psychoeducation (50). In the long term (7 years), readmission rates were lower in the psychoeducation group (54%) compared to the control group (88%) (24). Another study including 150 participants confirmed these results. People with schizophrenia participating in short psychoeducation programs for patients and family were also less often hospitalized over a 1-year period (50).

Several large-scale studies also confirmed the efficacy of psychoeducational family approaches. They showed that the interventions led to a 20% reduction in relapse rates with results being particularly clear for family interventions lasting over 3 months (53, 54). A recent review of 50 RCTs showed that family interventions were effective in various areas (55):

- Knowledge of the relatives about the disease;
- Reduced relapse rates after 2 years;
- Support and patient compliance

Psychosocial functioning was difficult to measure; the different ratings seem to support that hypothesis that family intervention does improve general functioning. Continuous data from the social functioning scale were in favor of the family intervention group, but doubts remain about the study’s robustness given the small number of participants (55). A review on psychoeducation quoted several studies investigating the impact of family psychoeducation on psychosocial functioning: it concluded that family interventions may have a significant impact on functional outcomes in patients with schizophrenia (on global and social functioning, social relationships, interest in obtaining a job, and management of social conflicts) and their families (on social contacts and perception of professional support) (50).

The effectiveness of family psychoeducation as an “evidence-based practice” has been established by several studies (54, 56, 57). Conclusions regarding hospitalization and relapse rates from randomized trials on family psychoeducation are reliable. Results are more contrasted as regards the alleviation of family burden (58). However, short-term psychoeducational interventions may still have positive effects on subjective burden, depression and anxiety, and could be especially useful for low expressed emotion families (59, 60).

### Efficacy of Cognitive-Behavioral Therapies

#### Efficacy of Social Skills Training

More than 23 controlled trials and several literature reviews have been published about the impact of social skills training. They show that patients with schizophrenia can learn a variety of skills (conversational, interpersonal problem-solving strategies, etc.) and that acquired skills are usually still present after 2 years (the maximum duration of the studies) (61–64).

In 2002, Pilling et al. conducted a meta-analysis of nine RCTs on social skills training and concluded that there was little evidence of benefit in any outcomes (63). However, the conclusion was contested by Mueser and Penn (64) and Bellack (62). Bellack reviewed four meta-analyses of skills training and concluded that social skills training has a significant effect on behavioral skills, social role functioning, and client satisfaction but not on symptom reduction and relapse (62). The results of several meta-analyses are consistent with these results. Pfammatter et al. (65) examined 19 controlled trials and found positive effects on social skills acquisition (ES = 0.77) and social functioning (ES = 0.39). However, they found only a mild effect (ES = 0.23) on relapse. Kurtz and Mueser (66) studied 22 RCTs including 1,521 patients with schizophrenia and found that skills training programs produce moderate but significant improvement in social functioning (ES = 0.52) and negative symptoms (ES = 0.40) and reduce hospitalization rates over a 1- to 2-year follow-up period (ES = 0.48–0.52). These results were consistently and sustainably maintained during the follow-up period. However, the effects of social skills training on other areas of psychopathology such as psychotic symptoms, relapse rates, and cognitive function are not consistent (65, 66). Two recent reviews on the treatment of negative symptoms in schizophrenia showed similar results. Five RCTs quoted in these reviews found that social skills training was associated with an improvement in negative symptoms. The gains were maintained after a 3- to 6-month follow-up period (67, 68). A recent meta-analysis also found social skills training to be superior to other interventions (69). Although social skills training was not initially conceptualized as a treatment for negative symptoms, these studies suggest that the technique could be effective for improving negative symptoms in the short term.

Conversely, the results of three Cochrane reviews (70–72) investigating life skills programs (teaching skills in budgeting, communication, domestic living, personal self-care, and community living) were contrasted and concluded that “compared to standard care, social skills training may improve the social skills of people with schizophrenia and reduce relapse rates but, at present, the evidence is very limited with data rated as very low quality.”

#### Efficacy of CT

There have been more than 40 controlled trials and several reviews on CT for psychosis, and most of them reached similar conclusions: CT is effective in reducing positive symptoms and improving social functioning (25, 27, 73). Several studies (74–77) also reported that the effects of CT were long-lasting (>1 year) and impacted positive symptoms. A meta-analysis (*n* = 33 studies) confirmed the positive effects of CT on positive symptoms with a moderate ES (ES = 0.37) but also showed its effectiveness on negative symptoms (ES = 0.44) and social functioning (ES = 0.38) (77). Granholm et al. (78) supported these results by studying 18 RCTs including measures of social functioning. Two-thirds of the studies showed significant improvement after CT, whereas the other meta-analyses reached less favorable conclusions (79–81). Later studies show a small ES on positive symptoms and little effect on relapse rate. The Lynch et al. study (79) was criticized for selecting works that did not specify the inclusion criteria, and for failing to monitor the effects in the selected studies. The studies were also criticized for the small size of the samples (approximately 600 patients) (82, 83). Several studies, published mostly in 2014, also seemed to support the efficacy of CT in reducing positive symptoms with an overall average ES (around 0.40). Recent meta-analyses highlighted the benefits of CT on both positive and persistent symptoms (77, 84, 85). The effectiveness of CT on negative symptoms seems less convincing. Meta-analyses using negative symptoms as a secondary outcome measure indicate that the effect of CT on negative symptoms is significant (77, 86). However, the moderate ES found in the first studies (77) is not as good in more recent studies (86). Also, only few studies have focused primarily on negative symptoms.

It seems that CT should comprise at least 20 sessions to be fully effective (87). Conversely, a recent meta-analysis showed that low intensity CT (fewer than 16 sessions) could have an effect on symptoms of psychosis (*d* = 0.46); these results were consistent with those found in other meta-analyses studying CT (88). In this meta-analysis, no significant between-group post-intervention differences were found for secondary outcome measures such as depression and anxiety or functioning; nevertheless, at follow-up, a statistically significant difference was observed between groups for depression and functioning. This may be an important finding as there could be delayed beneficial effects that may not always be seen immediately post-intervention (88).

Overall, CT is the most effective psychosocial intervention for psychotic symptoms while social skills training shows a modest but relatively robust effect on reducing negative symptoms compared to other psychosocial interventions (69).

Table 1 summarizes the main results of the studies with the largest samples of patients.

**Table 1:** Conclusions from meta-analyses including the largest samples of patients.

Psychosocial intervention	Meta-analyses	Description	Mains conclusions
Cognitive remediation	Wykes et al. (19)	40 Randomized controlled trials (RCTs), population with diagnosis of schizophrenia >70% (*n* = 2,104)	Cognitive remediation benefits people with schizophrenia and when combined with psychiatric rehabilitation, the benefit extends to functioning

Psychoeducation for patients	Xia et al. (47)	44 RCTs, patients with a diagnosis of schizophrenia or schizoaffective disorder (*n* = 5,122) (mostly inpatients)	Psychoeducation programs enhance treatment adherence, social functioning, and reduce relapse rates and readmission compared to standard care

Family psychoeducation	Pharoah et al. (55)	53 RCTs, patients with a diagnosis of schizophrenia or schizoaffective disorder (*n* > 4,800)	Family interventions decrease the frequency of relapses up to 2 years, and increase drug compliance, knowledge of the disease in the family, and reduce family burden

Social skills training	Kurtz and Mueser (66)	23 RCTs, patients with a diagnosis of schizophrenia or schizoaffective disorder (*n* = 1,521)	Large effect size (ES) for content learning and social skills, moderate ES for social functioning and negative symptoms

Cognitive therapy	Wykes et al. (77)	34 RCTs, patients with a diagnosis of schizophrenia or schizoaffective disorder (*n* = 1,964)	Moderate ES for global and positive symptoms (0.4). Effects inflated for less rigorous studies

## Discussion

Numerous results show that cognitive remediation, psychoeducation, and CBT are efficient rehabilitation tools. Data in the literature concerning cognitive remediation are homogeneous and show that it is efficient on cognitive functioning and psychosocial functioning, in particular the ability to work (20, 21, 23, 29). According to most studies, the impact of cognitive remediation on social functioning is more important both when combined with other rehabilitation techniques and when therapy is based on learning strategies (32, 19, 89).

Data on the effect of cognitive remediation on symptomatology are more heterogeneous. It probably has no effect on positive symptoms, and, in fact, severe positive symptoms can be an obstacle to improvement during cognitive remediation sessions. Data concerning negative symptoms are more complex since they are impacted by cognitive remediation. The effectiveness on negative symptoms is probably indirect, hypothetically due to a reduction of defeatist beliefs, avoidant behavior, and poor motivation, and, consequently, improvement in self-esteem (35).

Further studies should try to specify the effects of cognitive remediation, the active elements of interventions, the factors that lead to positive responses and the persistence of benefits over time (38). It seems, however, that factors such as motivation, social cognition, and metacognition may play a key role in the success of this remediation technique (20, 21).

Psychoeducation for families and patients proved to be effective in preventing relapses, readmission, and also in increasing drug compliance. Interventions with the highest level of evidence seem to be those involving relatives. Actually, psychoeducation for patients showed its effectiveness, but with a lower level of proof compared to patient and family psychoeducation (46). It is important for patient and family psychoeducation not only to transmit information but also to provide practical skills such as concrete problem-solving techniques.

Although methodological reductionism restricts psychosocial rehabilitation to a single intervention, it appears that interventions combining psychoeducation, cognitive and behavioral techniques, and homework strategies are more effective at increasing treatment adherence than unidimensional approaches (24, 90, 91). Future research should focus on the development of new kinds of programs such as peer-led psychoeducation. It seems essential for participants to receive information from and exchange with peers. Conversely, providing too much information about the disease can cause defensive reactions (50, 58). Uncertainties still remain about the efficiency of psychoeducation in areas such as global functioning, awareness of the disorder, need for care, and quality of life, especially in the long term (2 years) (22, 24). Other parameters need to be clarified by better designed studies, such as the minimum effective “dose” of psychoeducation and the specificity of the psychoeducational format according to patient status (50, 58).

Social skills training produced contradictory results. The lack of consistency is due to methodological problems in some studies including small samples, sampling biases, and lack of blinding to treatment allocation (18). However, there are few methodological issues with the Cochrane reviews, and many other studies are coming to the same conclusions. Social skills training was found to be efficient on social skills, on psychosocial functioning and on negative symptoms. With regard to more distal outcomes, existing reviews and meta-analyses do not consistently support the positive effects of social skills training on outcomes such as relapse rate, psychotic symptoms, and quality of life (18). Additionally, it has been found that various factors may influence the effectiveness of social skills training. For example, Mueser et al. (29) noted that deficits in attention may limit the effects of social skills training approaches. It also seems crucial to note that transferring the skills learned during therapy sessions to everyday life is not always easy, which is why generalization techniques (home-based exercises) are very important. They provide patients with the opportunity to practice skills in natural situations (18, 26, 28).

Social skills training has proved to be very efficient when associated with cognitive remediation or SE which is why the three rehabilitation interventions are often bundled (18, 92). Besides enabling patients to practice newly acquired skills in everyday life, it gives them appropriate feedback and provides social reinforcement (23).

The data in the literature concerning CT are quite homogeneous, indicating that CTs are efficient in reducing positive symptoms (73–77). Cognitive therapies may be used as adjuvants to chemotherapy in patients in remission or in patients with active symptoms and may also be effective in reducing negative symptoms. However, further controlled trials with negative symptoms as the primary outcome measure are required. The quality and effectiveness of cognitive therapies is partly determined by the training and the supervision of therapists (81). Additional studies on CT and minimal dosing are still required. Few works seem to show effectiveness of low intensity CBT, but low and high intensity CBT should be compared in future studies (88). It also seems important to consider for future research that patients do not always need their symptoms to be eradicated, and such observations are common in the literature on recovery from psychosis or schizophrenia. Recovery means being able to live with symptoms, i.e., being able to cope with the “voices.” Thus, although CT analyses focusing only on psychotic symptom reduction are important, further studies should focus on secondary outcomes such as reduced distress or self-defined recovery. We should also concentrate on changing how people relate to their thoughts and feelings, as the third-wave approaches do (30, 31).

In this review, we were particularly interested in the effect of the techniques on psychosocial functioning. The techniques that led to the most robust improvement in psychosocial functioning were cognitive remediation (32, 19) and socials skills training (63–66). With both techniques, improvement of social functioning depends a lot on a common characteristic that consists in supporting practice with rehabilitation activities (e.g., SE) or opportunities to reflect on how to apply the skills to everyday life. The programs also require frequent personal contact with a therapist. It is likely that by providing these elements, the programs facilitate learning consolidation by making new cognitive or social skills accessible in everyday life. Programs that use supported practice and other methods to maximize transfer of therapy-learned skills to everyday life and those involving a therapist may be more likely to have an impact on functioning (35, 67). A study showed the efficacy of CT on psychosocial functioning (77). Improvement in both positive and negative symptoms may lead to better functioning by limiting the consequences of the symptoms. The results suggest that there is a relationship between different outcomes and that targeting one outcome (e.g., positive symptoms) may have positive effects on others (e.g., functioning) (77). Regarding patient or family psychoeducation, the effect on psychosocial functioning seems limited, but that does not mean that the interventions have no effect on functioning, but rather that functioning assessments are rarely reported in works about psychoeducation intervention and when they are, functioning is not a priority outcome. Further research should investigate the effect of psychoeducation on functioning as a primary outcome measure. Since psychoeducation seems to be effective on variables influencing real-world functioning (engagement in service and internalized stigma), interpreting results on functioning were rather difficult because psychosocial functioning assessment is very heterogeneous in the literature. Most of the works reviewed here included studies using different scales. It seems that future research on psychosocial interventions could focus more on functional outcomes. Another important issue is how to assess real-world functioning: it would be useful to find a common set of criteria that would enable its assessment.

All these interventions are always delivered within the framework of rehabilitation and are not intended to be stand-alone treatments. Several programs combining interventions proved to be efficient, such as CBT and skills training, SE and skills training, cognitive remediation and social skills training (i.e., integrated psychological therapy) (92, 93), or social cognitive training and CBT and skills training (i.e., social cognition and interaction training).

The impact of psychosocial interventions on functional outcomes seems to be improved by combining elements from each therapeutic approach (16, 17, 94, 95). Clinical experience showed the relevance of combining techniques based on patient issues and the stage of the disease. These techniques seem to be complementary: on the one hand, psychoeducation and CBT allow patients to gain knowledge about their illness and play an active role in the recovery process while on the other, social skills training and cognitive remediation may enhance adaptive skills. Nevertheless, further research is needed to identify the synergistic effects of combined interventions and the active ingredients of successful therapeutic modalities.

## Conclusion

Recovery from schizophrenia seems to depend partly on functional outcomes such as neurocognition, social cognition, negative symptoms, and functional capacity. It therefore appears essential to assess these variables for each patient and to develop efficient rehabilitation interventions. According to the literature, some psychosocial interventions have proven their effectiveness: cognitive remediation for reducing the impact of cognitive impairment, social skills training for reducing negative symptoms, psychoeducation for improving compliance and reducing relapses, and CT for reducing the intensity of or distress related to positive symptoms. In addition, the techniques also try to promote the recovery process by encouraging self-determination and active empowerment.

Care is organized according to these scientific data and the local environment. Rehabilitation structures should be organized so the interventions are accessible to the largest possible number of patients and so research may be coordinated on the therapeutic effects of psychiatric rehabilitation, as is already the case in some French regions (Auvergne-Rhône-Alpes and Nouvelle-Aquitaine in particular) (96). Structures such as these offer the most varied rehabilitation care facilities, but they remain experimental, and their effectiveness has yet to be evaluated.

## Author Contributions

NF has defined the organization of care and the development of the psychosocial intervention in France. LM drafted the paper and both the authors approved the final version.

## Conflict of Interest Statement

The authors declare that the research was conducted in the absence of any commercial or financial relationships that could be construed as a potential conflict of interest.
